# Endoplasmic reticulum stress and IRE-1 signaling cause apoptosis in colon cancer cells in response to andrographolide treatment

**DOI:** 10.18632/oncotarget.9180

**Published:** 2016-05-05

**Authors:** Aditi Banerjee, Hafiz Ahmed, Peixin Yang, Steven J. Czinn, Thomas G. Blanchard

**Affiliations:** ^1^ Department of Pediatrics, University of Maryland School of Medicine, Baltimore, Maryland, U.S.A; ^2^ GlycoMantra Inc, Baltimore, Maryland, U.S.A; ^3^ Department of Obstetrics, Gynecology, and Reproductive Sciences, University of Maryland School of Medicine, Baltimore, Maryland, U.S.A

**Keywords:** endoplasmic reticulum stress, unfolded protein response, apoptosis, chemotherapy, andrographolide

## Abstract

The plant metabolite andrographolide induces cell cycle arrest and apoptosis in cancer cells. The mechanism(s) by which andrographolide induces apoptosis however, have not been elucidated. The present study was performed to determine the molecular events that promote apoptosis in andrographolide treated cells using T84, HCT116 and COLO 205 colon cancer cell lines. Andrographolide was determined to limit colony formation and Ki67 expression, alter nuclear morphology, increase cytoplasmic histone-associated-DNA-fragments, and increase cleaved caspase-3 levels. Andrographolide also induced significantly higher expression of endoplasmic reticulum (ER) stress proteins GRP-78 and IRE-1 by 48 h but not PERK or ATF6. Apoptosis signaling molecules BAX, spliced XBP-1 and CHOP were also significantly increased. Moreover, chemical inhibition of ER stress or IRE-1 depletion with siRNA in andrographolide treated cells significantly limited expression of IRE-1 and CHOP as determined by immunofluorescence staining, real time PCR, or immunobloting. This was accompanied by a decreased BAX/Bcl-2 ratio. Andrographolide significantly promotes cancer cell death compared to normal cells. These data demonstrate that andrographolide associated ER stress contributes to apoptosis through the activation of a pro-apoptotic GRP-78/IRE-1/XBP-1/CHOP signaling pathway.

## INTRODUCTION

Colorectal cancer (CRC) is the third most diagnosed cancer and the third leading cause of death due to cancer in the United States. The lifetime risk for developing CRC is about 1 in 20 (5%). [1] Chemotherapy plays an important role in the management of CRC. However, most chemotherapy agents have undesirable side effects including nausea, diarrhea, decreased blood cell counts, fatigue, nerve damage, pain and others. [2, 3] For these reasons as well as for increased efficacy, chemotherapeutic agents continue to be developed. One potential source for novel chemotherapy compounds are naturally occurring products in plants, particularly those with a history of medicinal use in many ethnic cultures.

Plant metabolites may constitute a vast source of potential new drugs and therefore many natural food components have been screened for protective or anticancer activities. [4] The diterpenoid lactone andrographolide is the major bioactive component isolated from Andrographis paniculata, a medicinal plant well documented to possess several pharmacologic activities, with its anti-inflammatory activity most extensively studied. [5–7] Andrographolide and its derivatives however, have also been reported to possess anti-cancer activity against several different types of cancer cells through inhibiting cell cycle progression, inducing apoptosis, and reducing cell invasion. [8–10] Most recent studies indicate that andrographolide acts as a strong radiosensitizer in human ovarian SKOV3 xenografts *in vivo* by increasing the BAX/Bcl-2 protein ratio and promoting the activation of caspase-3, leading to enhanced apoptosis as well as autophagy. [11] While there are many reports describing it's potent activity in inducing apoptosis in various cancer cell lines, the cellular mechanism(s) by which andrographolide induces apoptosis activity have not been elucidated. [12–15] One potential pathway is the induction of endoplasmic reticulum (ER) stress.

When protein folding in the ER is altered due to disturbances in redox, Ca^++^ levels, glycosylation or other environmental elements resulting in accumulation of misfolded proteins, eukaryotic cells activate a series of signal transduction cascades that are collectively termed the unfolded protein response (UPR). The hallmark of the UPR is the expression of ER-resident chaperones, such as GRP-78. In addition PERK, IRE-1 and ATF-6 serve as proximal sensors that regulate components which to upregulate the capacity of the ER to fold newly synthesized proteins and degrade misfolded/unfolded proteins. Activation of IRE-1 induces X-box binding protein 1 (XBP-1) mRNA splicing to generate the active form of the XBP1 transcription factor. These sensors can also serve as initiation points for the activation of signaling pathways that ultimately promote proapoptotic transcription factors leading to apoptotic cell death. We now report that andrographolide induces ER stress in cancer cells including activation of IRE-1, and that these events contribute to andrographolide associated cell death.

## RESULTS

### Andrographolide inhibits cell viability

**Figure 1:**
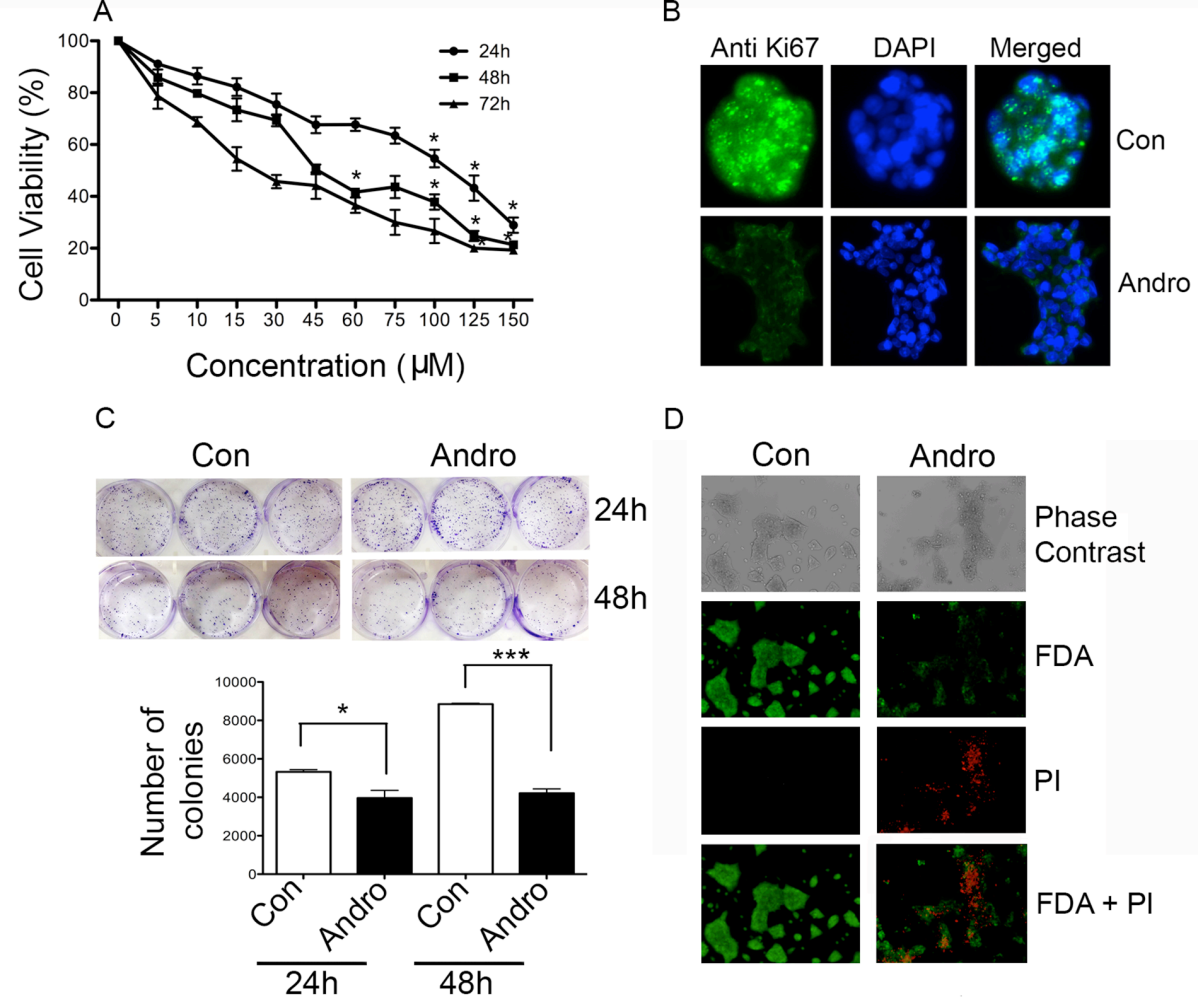
Andrographolide suppresses cell proliferation and clonogenicity in T84 cells **A.** Cells were treated with andrographolide for 24, 48 and 72 h and cell viability was quantified by MTT assay. **B.** T84 cells were stained with FITC labeled anti-Ki67 antibodies and DAPI and evaluated by fluorescence microscopy. **C.** T84 cells were diluted and treated with andrographolide at IC_50_. Growth was measured by direct counting of clonal clusters stained in multiwell plates with crystal violet at 24 and 48 h. Representative photomicrographs are shown. **D.** Fluorescence microscopy images showing the viability of T84 cells cultured *in vitro* with or without andrographolide (from top to bottom: phase contrast image, FDA staining, PI staining, composite of FDA and PI staining). (scale bar: 200 μm). Experiments were performed either two times (B and C) or three times (A and D). (*P < 0.05, **P < 0.01.)

T84 cells were treated with andrographolide (0-150 μM) for 24, 48 and 72 h to assess its effect on cell proliferation. MTT assays revealed significantly reduced cell viability in a time and dose dependent manner (Figure 1A). The IC_50_ was determined to be 45 μM at 48 h and this concentration was used for subsequent assays. Immunofluorescence staining for Ki-67 expression was evaluated to measure the effect of andrographolide on cell growth. Ki-67 was greatly reduced compared to untreated cells (Figure 1B). The inhibitory properties of andrographolide on T84 cells were also determined in a clonogenic assay and direct enumeration of stained colonies (Figure 1C). Treatment of cells for 24 or 48 h resulted in significantly fewer colonies compared with the untreated cells. The number of colonies decreased approximately 50% by 48 h (p<0.05). Viability of the cells was also visualized using FDA/PI double staining (Figure 1D). Andrographolide treated cells incorporated less FDA and increased PI indicating increased cell death.

### Andrographolide induces apoptosis in colon cancer cells

**Figure 2:**
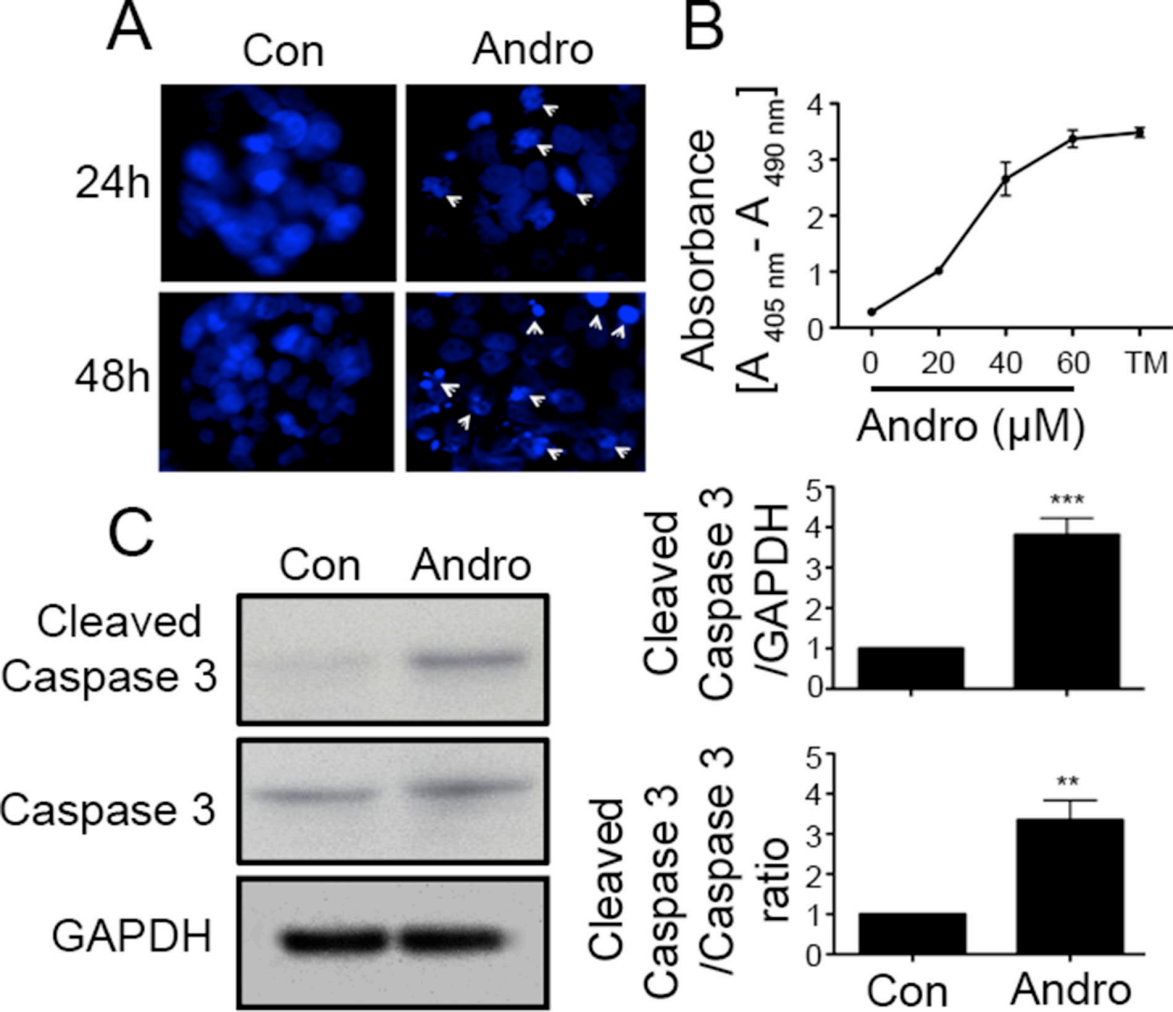
Andrographolide induces cell apoptosis in colon cancer T84 cells **A.** Cells were treated with andrographolide IC_50_ dose for either 24 (upper panels) or 48 h (lower panels) and stained with DAPI. Apoptotic cells were identified by condensation and fragmentation (arrows) of nuclei using inverted fluorescence microscope. **B.** Detection of nucleosomes in cytoplasmic fractions at increasing doses of andrographolide and TM. 10^4^ cells were treated with or without andrographolide (20, 40, or 60 μM) and TM for 48 h at 37°C. 20 μl of cell lysates were analyzed in the ELISA. **C.** Cleaved caspase-3 and caspase-3 protein expression was evaluated by immunoblotting of T84 cell lysates after 48 h of andrographolide IC_50_ treatment. Staining was normalized using GAPDH expression and the ratio of cleaved caspase and caspase-3 and amount of relative staining was determined by densitometry. (**P < 0.01, *** *P* < 0.001)

Andrographolide treated cells were examined using DAPI nuclear staining to assess whether loss in cell viability is also associated with apoptosis. As shown in Figure 2A, there was prominent nuclear fragmentation and chromatin condensation in andrographolide treated cells by 48 h of treatment (Figure 2A). Apoptotic cell death was also observed when quantifying cytoplasmic nucleosomes in andrographolide treated cells which increased in a dose dependent manner (Figure 2B). Additionally we measured the molecular alteration of apoptosis related proteins by western blot in andrographolide treated cells (Figure 2C). Andrographolide treatment increased the 17 kDa cleaved Caspase 3 levels at 48 h compared to control untreated cells (*P* < 0.001). The ratio of cleaved caspase 3 and total caspase −3 also significantly increased (*P* < 0.01).

### Andrographolide induces ER stress and associated pro-apoptosis signaling

**Figure 3:**
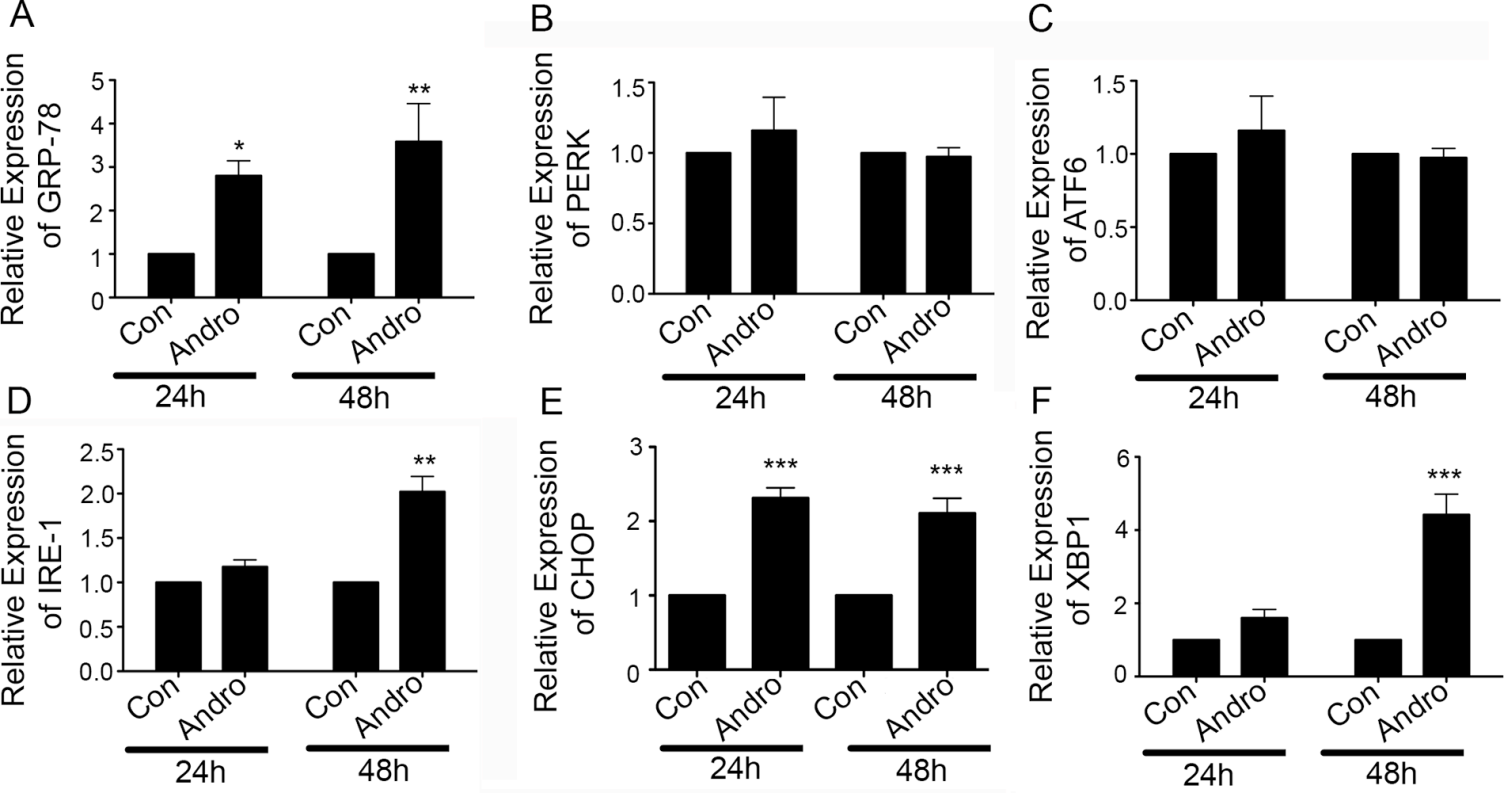
Andrographolide induces ER stress-related IRE-1 and associated proteins T84 cells were treated with Andrographolide at IC_50_ (45 μM) for 24 and 48 h and the transcriptional level of expression for ER Stress and apoptosis associated genes was determined by qRT-PCR for **A.** GRP-78, **B.** PERK, **C.** ATF6, **D.** IRE1, **E.** CHOP, and **F.** XBP-1. Bar graphs show quantitative results normalized to GAPDH mRNA levels. Results are from three independent experiments. Statistical significance was determined using one way-ANOVA followed by Bonferroni test. (*P < 0.05, **P < 0.01, ***P < 0.001)

One mechanism of inducing apoptosis is through activation of the UPR via ER stress. Therefore, andrographolide-treated T84 cells were examined for expression of the UPR marker, GRP-78. GRP-78 mRNA expression in treated cells was increased by ~2.5 fold and 3.5 fold at 24 and 48 h respectively (Figure 3A; *P* < 0.5, *P* < 0.001). Additional analysis was performed on the three UPR signaling pathway initiators PERK, IRE-1 and ATF6. Treatment resulted in a significant increase in IRE-1-α mRNA expression (~2 fold, *P* < 0.001) at 48 h (Figure 3D) but not for PERK or ATF6 (Figure 3B and 3C). Consistent with IRE-1 activation, an increase in XBP-1 mRNA of over 4.5 fold was also observed at 48 h (Figure 3E; *P* < 0.001). This increase in XBP-1 was also accompanied by a significant increase in spliced XBP-1 at both 24 and 48 h, a significant change in form that promotes apoptosis (Supplementary Figure 1A and 1B). Expression of CHOP, which can be activated by XBP-1, was also significantly increased (> 2 fold) but this expression was noted at 24 h of andrographolide stimulation (Figure 3F, *P* = 0.0015).

### Andrographolide induced apoptosis signaling is dependent upon ER stress

**Figure 4:**
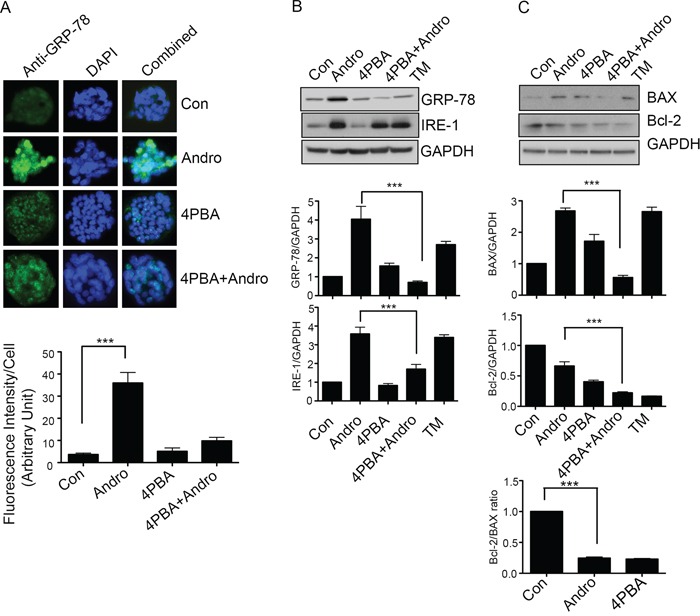
Andrographolide induced apoptosis signaling is dependent on ER Stress **A.** T84 cells were grown on coverslips and treated with Andrographolide IC_50_ in the presence or absence of 4-PBA and GRP-78 expression was evaluated by immunofluorescent staining. Nuclei were stained using DAPI and cells were examined by fluorescence microscopy. Fluorescence intensity was determined and compared with untreated T84 cells. **B.** T84 cells treated with Andrographolide IC_50_ for 48 h were lysed and protein expression was determined by immunoblotting for GRP-78, IRE1, and GAPDH. Densitometry analysis was performed and normalized with GAPDH expression to demonstrate significant reductions in expression of GRP-78, IRE1, in the present of 4-PBA. **C.** Cell lysates were analyzed for pro-apoptotic BAX and anti-apoptotic Bcl-2 expression by immunoblot analysis and quantified by densitometry. The lower graph shows the ratio of Bcl-2/BAX. The results shown are from three independent experiments. (***P < 0.001)

T84 cells were treated with andrographolide in the presence of 4-PBA to block ER stress. Cells were also treated with tunicamycin (TM) as a positive control for induction of ER stress and apoptosis. As shown in Figure 4A, andrographolide induced strong expression of GRP-78 by immunofluorescence (*P* < 0.001) but levels returned to background in the presence of 4-PBA. Western blot analysis confirmed a significant reduction in GRP-78 as well as IRE-1 expression in the presence of 4-PBA (Figure 4B, *P* < 0.001). The expression levels of both Bcl-2 and Bax were also analyzed by Western blot as Bcl-2 family proteins are important regulators of apoptosis. [25, 26] Andrographolide treatment upregulated BAX expression, a pro-apoptotic protein but not expression of Bcl-2, an anti-apoptotic protein (Figure 4C). The Bcl-2/BAX ratio was significantly decreased in andrographolide-treated T84 cells compared to untreated cells. The presence of 4-PBA however significantly reduced the expression of BAX protein to levels observed in the absence of andrographolide (*P* < 0.001).

Analysis was also performed on additional colon cancer cell lines to demonstrate that our observed andrographolide associated activity was common to colon cancer cells and not a specific characteristic of T84 cells. HCT 116 cells were stimulated with andrographolide at the published IC_50_ value. [18] Andro significantly increased gene expression of GRP-78 (~3 fold), IRE-1 (~2 fold), PERK (~2.5 fold), ATF-6 (~3.4 fold) and CHOP (~6 fold) compared to control cells and GRP78 and CHOP expression was greater that observed when stimulating with TM as a positive control (Supplementary Figure 2). Western blot analysis on ER stress proteins however revealed increases only in GRP78 and IRE1 expression, consistent with T84 results. Treatment of andrographolide stimulated cells with 4-PBA showed significant decreases in these proteins comparable to untreated controls. These results were consistent for both gene expression and western blot. A third colon cancer cell line COLO 205 was also evaluated following treatment with andrographolide and expression of ER stress response and pro-apoptosis genes as measured by RT-PCR were consistent with the results obtained with T84 and HCT 116 cells (data not shown).

### Andrographolide does not activate other ER stress sensors or MAPK

**Figure 5:**
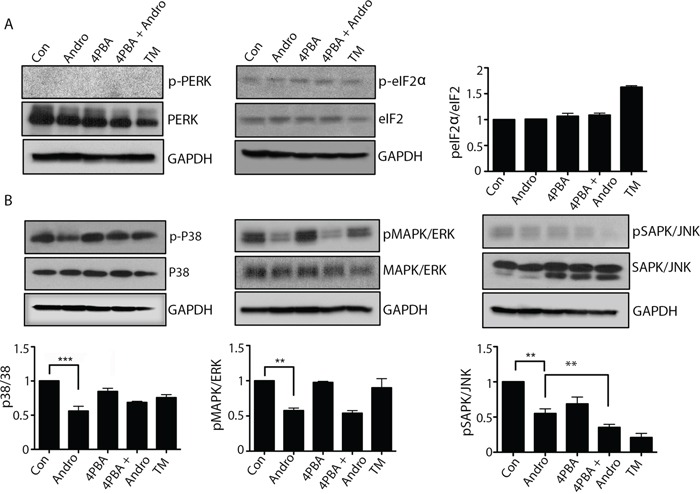
Andrographolide induced ER stress does not activate PERK or ATF-6 pathways, and downregulates MAPK pathways T84 cells were treated with Andrographolide at IC_50_ (45 μM) for 24 and 48 h and evaluated by western blot for **A.** phospho-PERK, phospho-eIF2α and GAPDH or **B.** phospho-p38, phospho-ERK1/2, and phospho-SAPK/JNK and quantified by densitometry. The results shown are from three independent experiments. (**P < 0.001)

The downstream activity of other ER stress pathways was also evaluated. Andrographolide did not induce phosphorylation of either PERK or eIF-2α (Figure 5A) The phosphorylation of MAPKs was investigated as well since ER stress has been shown to influence kinase activation. [19] Andrographolide treatment resulted in a significant reduction in phospho-p38, phospho-ERK1/2, and phospho-JNK (Figure 5B). The addition of 4PBA in combination with andrographolide did not reverse the inhibition of phosphorylation of these kinases.

### Andrographolide mediated apoptosis is dependent on IRE-1

**Figure 6:**
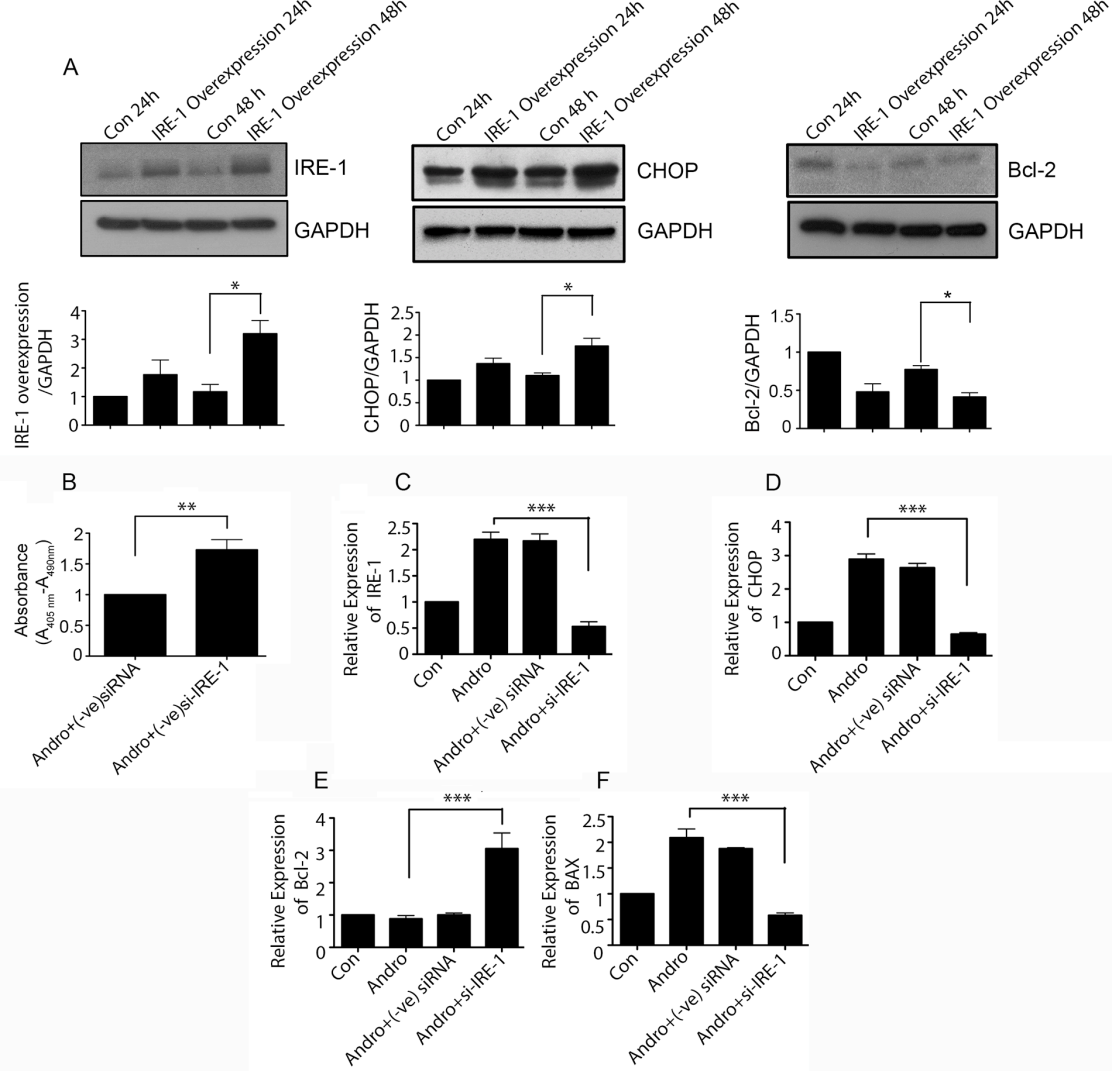
Andrographolide induced apoptosis signaling is dependent on IRE-1 **A.** T84 cells were transfected with plasmid for overexpression of IRE-1 and then treated with Andrographolide. Cell lysates were analyzed by western blot and quantified by densitometry for expression of IRE-1, CHOP, and Bcl-2. Expression is normalized against GAPDH expression. T84 cells were transfected with siRNA for IRE-1 or control siRNA and treated with Andrographolide for 48 h and **B.** DNA fragmentation was compared using a colorimetric assay. (C) Cells were also evaluated for mRNA expression by qRT-PCR for **C.** IRE-1, **D.** CHOP, **E.** Bcl-2, and **F.** BAX. (***P < 0.001)

To determine the nature of IRE-1 activity in andrographolide mediated apoptosis, IRE-1 was overexpressed in T84 cells with maximum expression observed at 48h (p<0.05) (Figure 6A). Overexpression of IRE-1 resulted in increased levels of CHOP (middle panel) and a decrease in Bcl-2 (right panel) at 48 h (*P* < 0.05). Cells were also evaluated when IRE-1 was depleted with siRNA. As shown in Figure 6B, IRE-1 depletion increased cell viability almost two fold in andrographolide treated cells. Transfection with IRE-1 siRNA resulted in significantly reduced IRE-1 mRNA levels compared to andrographolide treated cells transfected with control siRNA (Figure 6C, *P* < 0.001), as well as significantly less CHOP (Figure 6D, *P* < 0.001), and BAX (Figure 6F, *P* < 0.001). Expression of anti-apoptosis marker Bcl-2 however, was upregulated in andrographolide stimulated cells transfected with IRE-1 siRNA compared to negative controls (Figure 6E, *P* < 0.001). IRE-1 knockdown also resulted in a significant decrease in spliced XBP-1 levels (Supplementary Figure 1C). Taken together, the data indicate that andrographolide induced apoptosis is mediated via ER stress and the IRE-1 activation pathway.

### Growth inhibition properties of andrographolide are increased in colon cancer cells compared to non-cancer cells

**Figure 7:**
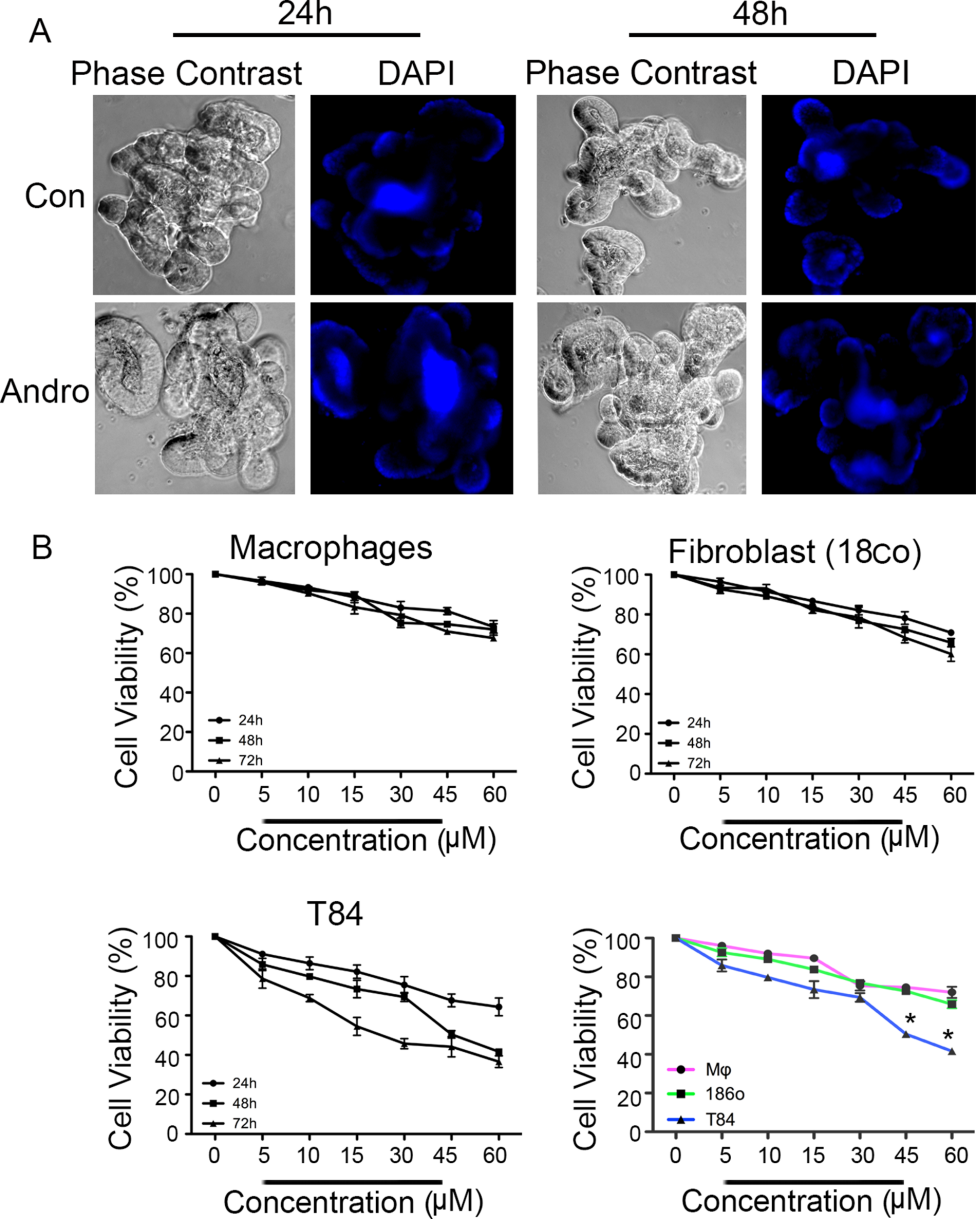
Andrographolide associated reductions in viability of are less pronounced in noncancer cells **A.** Three dimensional primary cultures of mouse intestinal epithelial cells organoids were expanded and treated with Andrographolide IC_50_ for 24 and 48 h and then stained with DAPI to evaluate nuclear morphology. **B.** Primary cultures of mouse bone marrow macrophages and human fibroblasts were treated with Andrographolide at the indicated dose range and compared to Andrographolide treated T84 cells for cell viability using the MTT assay. The data are expressed as the percentage of viable cells relative to untreated control cells. Data shown are from three independent experiments. (P < 0.05)

Optimal use as a chemotherapeutic agent would require heightened pro-apoptotic activity against cancer cells compared to noncancerous cells. Several distinct cell types were treated with andrographolide to compare its activity. Three dimensional mouse intestinal epithelial cell organoids were prepared as a source of expanding non-cancerous gut epithelial cells. Organoids were treated with andrographolide for either 24 or 48 h, similar to T84 cells and then fixed for nuclear DAPI staining (Figure 7A). Organoids retained their typical multi-lobed character and the integrity of the monolayers appeared normal. DAPI staining failed to reveal any nuclear fragmentation even at 48 h of treatment. Additional analysis was performed on primary cultures of mouse macrophages and a nontransformed human gut fibroblast cell line (Figure 7B). Cell proliferation of these cells, as well as T84 cells, were determined in the presence of varying concentrations of andrographolide (5 – 60 μM) at 24, 48, and 72 h. T84 cells dropped to 50 and 40% viability in the presence of 45 and 60 μM respectively at 48 h whereas macrophages and fibroblasts retained approximately 70% viability at these concentrations at 48 h, andrographolide therefore induces significantly less cell viability in colon cancer cells compared to either cell line (*P* < 0.05).

## DISCUSSION

The present study demonstrates the induction of ER stress-related proteins may be involved in andrographolide induced apoptosis. The ability of cells to respond to perturbations in ER function, or ‘ER stress’, is critical for cell survival, but chronic or unresolved ER stress can lead to apoptosis. [20] In multicellular eukaryotes, ER stress is sensed by three upstream signaling proteins that begin a cascade of corrective actions. The activity of these three pathways collectively constitutes an ER-specific UPR. [21] High level or prolonged activation of theUPR signaling is related to cell death. Thus, the degree and duration of the UPR evaluated by sensors of ER stress (IRE-1, PERK and ATF6) seems to be critical for cell death or survival. We screened these UPR sensors as well as downstream signaling molecules and measured their intensity after treatment of T84, HCT 116, and COLO 205 colon cancer cells with andrographolide. Activation of UPR proteins at 48 h was associated with increased pro-apoptosis signaling and cell death.

Andrographolide induced GRP-78 and CHOP expression. CHOP is one of the highest inducible genes during ER stress. Andrographolide induced IRE-1 expression, one the three major ER stress activated UPR proteins. Although increases in message for ATF6 and PERK were observed in HCT 116 cells, only IRE-1 demonstrated increases in protein levels following andrographolide treatment, an observation that was consistent among all three cell lines. Andrographolide also induced splicing of XBP1 mRNA. Under ER stress conditions, IRE-1 catalyzes the excision of an intron in XBP1 mRNA. The modified spliced mRNA product then produces the functional pro-apoptosis XBP1 transcription factor. [22, 23]

It is notable that transcriptional levels of CHOP were observed at 24 h even though GRP-78 and IRE-1 transcriptional expression was not significantly increased until 48 h. It is difficult to determine which of the three main UPR proteins might be driving transcriptional expression of CHOP at 24 h as neither IRE-1, PERK or ATF6 were significantly increased at this time point although all three did show slight elevations at 24 h. IRE-1 however was the only protein to continue to increase in expression and achieve significance at 48 h, a time point at which we observed significant increases in CHOP protein by immunoblot.

It is also noteworthy that while andrographolide induced ER stress mediated apoptosis in the T84 colon cancer cell line, it had significantly less cytotoxicity in normal human gut Fibroblasts, mouse macrophages, or mouse intestinal epithelial cell organoids. These data suggest andrographolide is more efficient at inducing ER stress and apoptosis in cancer cells than in nontransfomed gut epithelial cells or noncancerous resident cells such as fibroblasts and macrophages. The resistance of noncancerous cells to andrographolide associated apoptosis will require additional study but this characteristic strengthens the rationale the use of andrographolide as a chemotherapeutic agent.

In summary, these results demonstrate that andrographolide induces apoptosis in colon cancer cell lines and that ER stress and the UPR acting primarily through activation of IRE-1 are significant events in andrographolide induced cell death. This is the first report to demonstrate the ability of andrographolide to induce ER stress as contributing element in the induction of apoptosis. The manner in which andrographolide promotes ER stress in cancer cells however is not clear. Therefore, further investigation on andrographolide will be necessary to delineate its molecular interactions in cells and how these interactions induce ER stress.

## MATERIALS AND METHODS

### Mice

C57BL/6 mice (Jackson Laboratory, Bar Harbor, ME) were housed under pathogen-free conditions in microisolater cages at the University of Maryland Baltimore animal facilities. This study was carried out in strict accordance with the Guide for the Care and Use of Laboratory Animals of the National Institutes of Health. The protocols were approved by the Institutional Animal Care and Use Committee of the University of Maryland in Baltimore.

### Cells

The T84, HCT 116 human colon cancer cell line (ATCC^®^ CCL-248™) and the CCD-18Co human fibroblast cell line (ATCC^®^ CRL-1459™) were purchased from the American Type Culture Collection (Manassas, VA). The T84 cells and HCT 116 cells were grown in DMEM/F-12 and RPMI-1640 nutrient media with 5% and 10% FBS respectively, and CCD-18Co cells were cultured in DMEM with 10% FBS and a 1 to 100 dilution of 100X stock MEM nonessential amino acids as previously described. [24] Bone marrow derived macrophages (BMDM) were derived from mice as previously described [17] and cultured in complete RPMI media supplemented with 10% FBS and 20% conditioned media from LADMAC cells as a source of CSF-1. [26] All media were supplemented with a 1X solution of antimicrobial reagents (10,000 U/ml penicillin, 10,000 μg/ml streptomycin, 25 μg/ml amphotericin B).

### Three dimensional intestinal epithelial organoids

Crypt isolation and organoid cultures from murine small bowel were performed based on the methods of Sato et al [27] and using the Intesticult™ system from StemCell Technologies (Vancouver, Canada) according to manufacturer's instructions.

### ER stress induction in epithelial cells

T84 cells (70 – 80% confluent) were incubated with serum free DMEM:F12K containing one third the concentration of antimicrobials (3,330 U/ml penicillin, 3,330 μg/ml streptomycin, 8.3 μg/ml amphotericin B) for 3 h. Media was replaced with DMEM:F12K medium containing 2% FBS in the presence of andrographolide (Sigma Aldrich, St. Louis, MO) at IC_50_ for 24 and 48 h. ER stress was blocked in some experiments by adding 4-PBA (Sigma Aldrich) to the cells 30 min prior to andrographolide treatment. In some experiments, cells were treated with 1 μg/ml tunicamycin (TM) in parallel as a positive control for ER stress and apoptosis. [28]

### Cytotoxicity assay

Cytotoxicity was assessed using the MTT assay (Sigma Aldrich) as previously described. [29] Briefly, cells were plated in phenol red-free media in the presence of the indicated dose of andrographolide. The media was replaced with MTT solution at either 24, 48, or 72 h followed by the addition of DMSO to dissolve formazan crystals. Absorbance at 570 nm was determined using a microplate reader (Model 550, Bio-Rad, USA). The 50% inhibitory concentration (IC_50_) was the concentration of andrographolide that caused a 50% decrease in the optical density compared to untreated cells.

### Viability assay

Cell viability was determined by fluorescein diacetate (FDA; F7378, Sigma) /propidium iodide (PI; P4170, Sigma) staining according to manufacturer's instructions (Ibidi, Madison, WI). Images were taken using a fluorescence microscope.

### Clonogenic assay

T84 cells seeded in 6 well plates (5×10^5^/well) for 24 h were gently washed with PBS and then incubated with media containing andrographolide for 24 and 48 h. Cells were trypsinized, washed, and then seeded in 6-well plates (5×10^3^ cells/well) for 14 days. Cells were fixed in the 6-well plates with 10% buffered formalin solution for 15 min, and then stained with 2 ml 0.01% (w/v) crystal violet in dH_2_O for 30 min. Wells were washed with dH_2_O and dried. Colonies were counted manually using an inverted microscope.

### DAPI staining

T84 cells or intestinal organoids were evaluated by staining with DAPI (Vector Laboratories; Burlingame, CA) according to the method described by Majumdar et al. [29]

### Apoptosis assay

Specific determination of mono-and oligonucleosomes in the cytoplasmic fraction of cells treated with different concentrations of andrographolide for 48 h and siRNA was assessed using the Cell Death Detection ELISA kit (Roche Applied Science) according to manufacturer's instructions.

### SDS-PAGE and immunoblot

SDS-PAGE and western blotting were performed as previously described. [28] Briefly, cell lysates were generated using RIPA buffer containing protease phosphatase inhibitor cocktail (Pierce). Cell lysates were subject to centrifugation and the protein content in supernatants was determined using the BCA Protein Assay Kit (Pierce). Proteins were resolved by 7.5%-12% SDS-PAGE, transferred to PVDF membrane and blocked with 5% bovine serum albumin. Proteins were detected with the following antibodies; cleaved Caspase 3 (MAB835) and hGRP-78 (MAB4846) from R&D Systems, BAX (ab32503) and Bcl-2 (ab7973) from Abcam, GAPDH (G8795) from Sigma Aldrich, p-PERK (sc-32577) from Santa Cruz Biotechnology, and Caspase-3 (9662), IRE-1 (3294S), CHOP (2895S), p-eIF2α (3597s), eIF2 (9722), PERK (3192S), p-P38 (9211), P38 (9212), p-Erk1/2 (9101), Erk (9102), p-SAPK-JNK (9251), and SAPK/JNK (9252) from Cell Signaling. ATF6 (MA5-16172) was purchased from Thermo Fisher Scientific. Blots were incubated with HRP-conjugated secondary antibodies followed by enhanced chemiluminescence (ECL) detection. Results were quantified by densitometry of digitized images using ImageJ software (NIH, Bethesda, MD, ver.1.43) and expressed as a ratio to a GAPDH loading control.

### RT-PCR analysis

Relative expression of XBP-1/XBP-1s within a sample was determined using the XBP-1 cDNA fragment amplified with XBP-1s primers (Supplementary Table 1). GAPDH expression was used as an internal control. The cycling conditions were as follows: 2 min at 94°C, 15 s at 94°C, 1 min at 60°C, and 30 s at 72°C. The number of PCR amplification cycle of XBP-1 and GAPDH were 35 and 25 respectively. PCR products were detected on 3% agarose gels.

### Quantitative real-time polymerase chain reaction (qRT-PCR)

Gene expression was evaluated as previously described. [30] Briefly, RNA isolated from cells was converted to cDNA using the Quantitect Reverse Transcription kit (Qiagen). The cDNA was diluted 10-fold and PCR amplification was performed with an Eppendorf Realplex Instrument (Eppendorf AG, Hamburg, Germany) with SYBR Green supermix (Fermentas, Glen Burnie, MD), 0.8 μM of each primer, and 1 μl cDNA. Samples were run in triplicate. Primer sequences are listed in Supplementary Table 1. Relative gene expression changes were calculated using the 2^−ΔΔCT^ method, and expression normalization was accomplished using housekeeping gene glyceraldehydes 3-phosphate dehydrogenase (GAPDH).

### IRE-1 overexpression and depletion

Plasmid for IRE-1 overexpression was purchased from Addgene (IRE-1 α-pcDNA3.EGFP, #13009). For IRE-1 depletion, siRNA specific for the IRE-1 gene and a proprietary universal negative control siRNA that does not target any known gene were purchased from Sigma Aldrich (#EHU002721 and #SIC001 respectively). The IRE-1 siRNA is a mixture of siRNA sequences prepared from an enzymatic digestion of IRE-1 cDNA. T84 cells were transfected with siRNA oligonucleotides using TurboFect (Thermo Scientific,#R0533) according to the manufacturer's recommendations. Briefly, log phase T84 cells (5 × 10^4^ cells/well in 24 well plates) were transfected with 1 μg siRNA in 100 μl serum free DMEM and transfection reagent and cells were analyzed for gene expression at 24 and 48 h post transfection. For activation experiments, transfected cells were treated with andrographolide for 48 h. IRE-1 overexpression or depletion was validated by qRT-PCR or Western blot.

### Immunofluorescence

T84 cells were grown on glass cover slips for 24 h and treated with andrographolide as described. Cells were fixed in ice-cold methanol for 5 min, permeabilized with 0.2% Triton X-100 for 10 min, blocked with 6% BSA for 30 min and then incubated with primary antibodies Ki67 (Abcam, # 15580,) and GRP-78 overnight at 4°C. Cells were then incubated with Alexa Flour 488 labeled goat anti-mouse IgG (H+L) (1:200) (Invitrogen) and DAPI for 1 h. [31, 32] Sample images were taken with 200X magnification.

### Fluorescence microscopy and image acquisition

Fluorescence microscopy and colony counting were performed using an inverted fluorescence microscope (Olympus IX-71) (Pennsylvania, USA). Image analysis was performed using Metamorph image analysis software (Molecular Devices, Sunnyvale, CA). Fluorescence intensity was quantified using ImageJ software version 1.39 (NIH). RGB composite images from control and andrographolide-treated cells were created using Axion Vision rel, 4.6 and analyzed. Cells from five different fields were used for statistical analysis as previously described. [20]

### Statistical analysis

Data are presented as mean ± standard Error (S.E). Statistical analysis was carried out with Graph Pad Prism for Macintosh 5.0c (Graph Pad Software Inc., San Diego, CA). The mean S.E. was calculated by one-way analysis of variance (ANOVA). Significance between groups was further analyzed using the post hoc Tukey's test and Bonferroni test. *P* values were considered significant is less than 0.05 and are indicated as throughout using asterisks * = *P* < 0.05, ** = *P* < 0.01, *** = *P* < 0.001.

## SUPPLEMENTARY FIGURES AND TABLE


