# Supplementary material for: Endoplasmic reticulum stress and IRE-1 signaling cause apoptosis in colon cancer cells in response to andrographolide treatment
Source: Oncotarget. 2016 May 5;7(27):41432–44. doi: 10.18632/oncotarget.9180 (PMC5173070; doi:10.18632/oncotarget.9180)
Supplement: Supplementary file 1 [file oncotarget-07-41432-s001.pdf]

SUPPLEMENTARY FIGURES AND TABLE

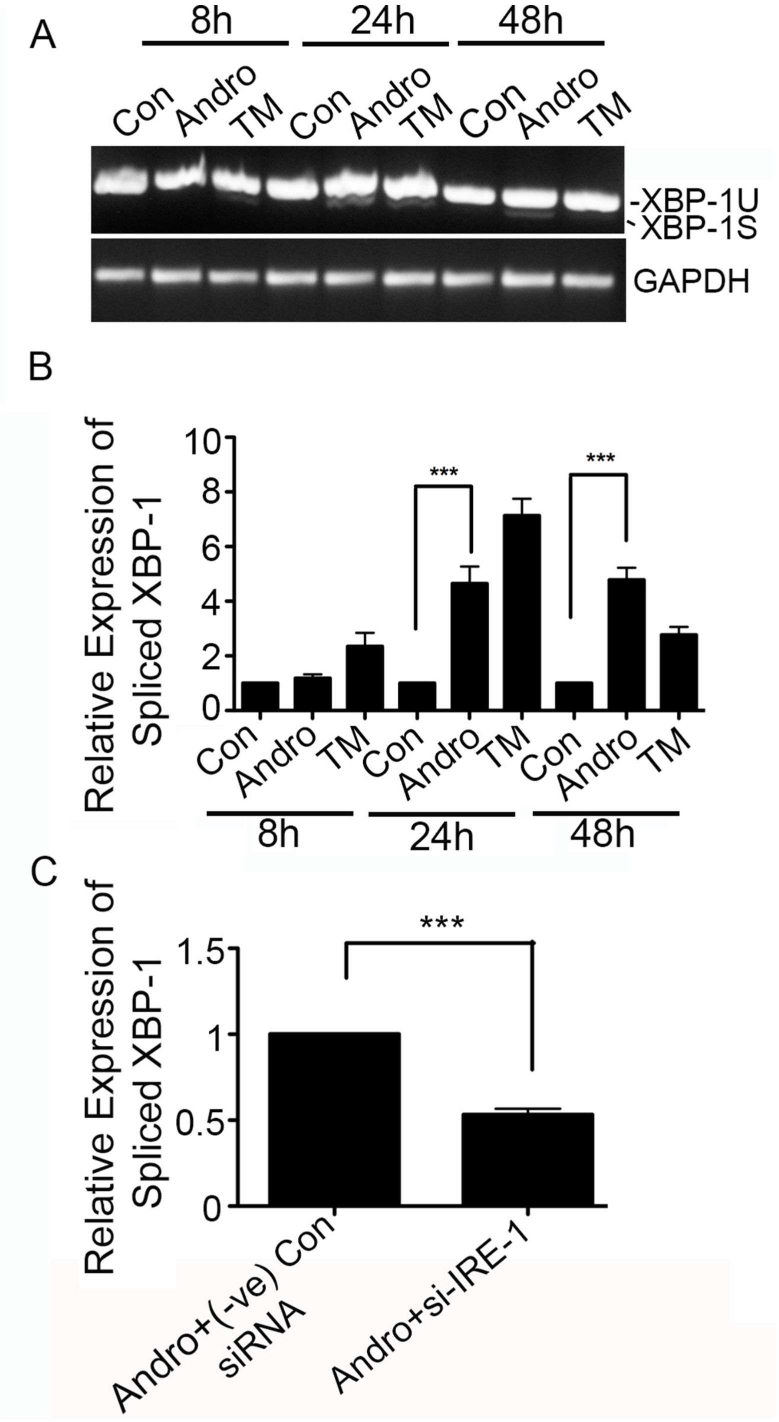

Supplementary Figure S1: Andrographolide stimulates XBP-1 splicing in T84 cells. A. T84 cells were treated with of andrographolide (45 μM) or TM (2 μg/ml) for the indicated time and RT-PCR was used to detect the presence spliced XBP-1 mRNA. The RT-PCR products were analyzed by 3.0% agarose gel electrophoresis. GAPDH was used as the internal control. **B.** T84 cells were treated with of andrographolide (45 μM) or TM (2 μg/ml) for the indicated time and the spliced form of XBP1 mRNA was analyzed by semi-quantitative RT-PCR. **C.** T84 cells were transfected with IRE-1 siRNA or control siRNA prior to treatment with andrographolide (45 μM) for 48 h and the spliced form of XBP1 mRNA was analyzed by semi-quantitative RT-PCR. (***P < 0.001)

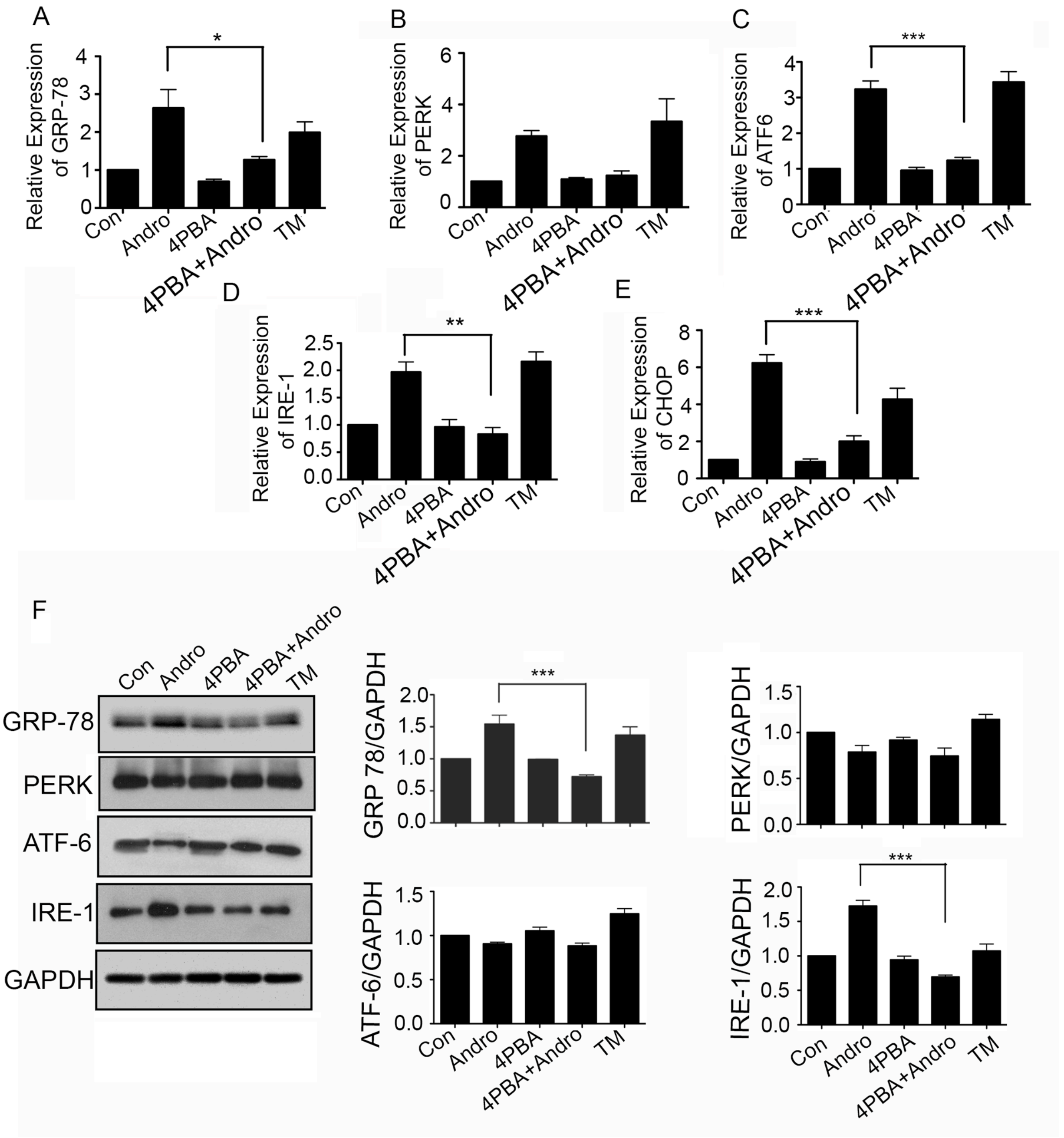

Supplementary Figure S2: Andrographolide induces ER stress-related proteins in HCT 116 colon cancer cells. HCT-116 cells were treated with andrograpaholide at IC_{50} (60 µM) for 24 h in the presence or absence of 4-PBA and TM. Transcriptional levels of expression for ER stress associated genes was monitored by qRT-PCR for **A.** GRP-78, **B.** PERK, **C.** ATF-6, **D.** IRE-1, or **E.** CHOP. Bar graphs show quantitative results normalized to GAPDH mRNA levels. Results are from three independent experiments. Statistical significance was determined using one way-ANOVA followed by Bonferroni test (P<0.05, **P<0.01, ***P<0.001). **F.** HCT-116 cell lysates were prepared with above mentioned conditions and protein expression was determined by immunoblot for GRP-78, PERK, ATF-6, IRE-1, and GAPDH. Densitometry analysis was performed and normalized with GAPDH.

Supplementary Table S1: qRT-PCR primers

Gene	Primer sequence forward	Primer sequence reverse
GAPDH	5′-CGACCACTTTGTCAAGCTCA-3′	5′-AGGGGAGATTCAGTGTGGTG-3′
GRP-78	5′-GGTGAAAGACCCCTGACAAA-3′	5′-GTCAGGCGATTCTGGTCATT-3′
ATF6	5′-TCAGGGAGTGAGCTACAAGT-3′	5′-CTTGTGGTCTTGTTATGGGT-3′
CHOP	5′-TTCTCTGGCTTGGCTGACTG-3′	5′-CTGCGTATGTGGGATTGAGG-3′
IRE-1	5′-GGGAAATACTCTACCAGCCT-3′	5′-GAAATCTCTCCAGCATCTTG-3′
PERK	5′-ATCCCCCATGGAACGACCTG-3′	5′-ACCCGCCAGGGACAAAAATG-3′
XBP1	5′-AACCAGGAGTTAAGACAGCGCTT-3′	5′-CTGCACCCTCTGCGGACT-3′
XBP1s	5′-GTTGAGAACCAGGAGTTAAGACAG-3′	5′-CAGAGGGTATCTCAAGACTAGG-3′
Bax	5′-GAGAGGTCTTTTTCCGAGTGG-3′	5′-CCTTGAGCACCAGTTTGCTG-3′
Bcl-2	5′-GGAGGCTGGGATGCCTTT3′	5′-ACCCATGGCGGTGACCATGC-3′
